# A Novel Lysolecithin Model for Visualizing Damage *in vivo* in the Larval Zebrafish Spinal Cord

**DOI:** 10.3389/fcell.2021.654583

**Published:** 2021-05-20

**Authors:** Angela D. Morris, Sarah Kucenas

**Affiliations:** ^1^Neuroscience Graduate Program, University of Virginia, Charlottesville, VA, United States; ^2^Department of Biology, University of Virginia, Charlottesville, VA, United States

**Keywords:** demyelination, oligodendrocyte, zebrafish, myelin, central nervous system, spinal cord

## Abstract

**Background:** Lysolecithin is commonly used to induce demyelinating lesions in the spinal cord and corpus callosum of mammalian models. Although these models and clinical patient samples are used to study neurodegenerative diseases, such as multiple sclerosis (MS), they do not allow for direct visualization of disease-related damage *in vivo*. To overcome this limitation, we created and characterized a focal lysolecithin injection model in zebrafish that allows us to investigate the temporal dynamics underlying lysolecithin-induced damage *in vivo*.

**Results:** We injected lysolecithin into 4–6 days post-fertilization (dpf) zebrafish larval spinal cords and, coupled with *in vivo*, time-lapse imaging, observed hallmarks consistent with mammalian models of lysolecithin-induced demyelination, including myelinating glial cell loss, myelin perturbations, axonal sparing, and debris clearance.

**Conclusion:** We have developed and characterized a lysolecithin injection model in zebrafish that allows us to investigate myelin damage in a living, vertebrate organism. This model may be a useful pre-clinical screening tool for investigating the safety and efficacy of novel therapeutic compounds that reduce damage and/or promote repair in neurodegenerative disorders, such as MS.

## Introduction

Multiple sclerosis (MS) is a neuroinflammatory disorder characterized by the inappropriate attack of the myelin sheath by the immune system, which results in demyelination, axonal degeneration, and eventually severe disability. MS primarily attacks the central nervous system (CNS) in the form of focal demyelinating lesions found within the brain, spinal cord, and/or optic nerve, and patients battle various neurological challenges that are unique to lesion location. Mammalian models commonly used to study MS, including experimental autoimmune encephalomyelitis (EAE) and drug-induced demyelination, as well as patient data or clinical samples, do not allow researchers to directly visualize processes underlying myelin destruction and repair *in vivo.*

Lysophosphatidylcholine, commonly referred to as lysolecithin, has been used in mammalian models, including mice, rats, rabbits, cats, and Macaque monkeys, to induce demyelinating lesions in the spinal cord and the corpus callosum (Blakemore et al., 1977; Dousset et al., 1995; Foote and Blakemore, 2005; Girard et al., 2005; Gregg et al., 2007; Blakemore and Franklin, 2008). Injections of 1% lysolecithin are sufficient to induce demyelination in the CNS of these animals, and evidence of remyelination begins between 7 and 10 days post-injection in rodents (dpi) (Jeffery and Blakemore, 1995; Kotter et al., 2001).

Although a generalized demyelination model exists in zebrafish that takes advantage of the ease of drug submersion combined with tissue-specific ablation by the bacterial nitroreductase gene, a major limitation of this model is the inability to create focal lesions, which are a hallmark of MS pathology (Chung et al., 2013; Fang et al., 2014; Karttunen et al., 2017; Karttunen and Lyons, 2019). A previous study has also demonstrated that 1% lysolecithin placed on gelatin foam can successfully induce demyelination when applied directly to the adult zebrafish optic nerve (Münzel et al., 2014). More recently, a model of lysolecithin injection into the trunk of larval zebrafish was reported that utilized *in vivo* imaging to describe the pro-inflammatory response after demyelination (Cunha et al., 2020). In this manuscript, we have also created and characterized a model that induces focal lesions in the zebrafish spinal cord, allowing us to visualize lysolecithin-induced damage in a living, intact vertebrate model system.

## Results

### Creating a Focal Lysolecithin Injection Model in Zebrafish

**FIGURE 1:**
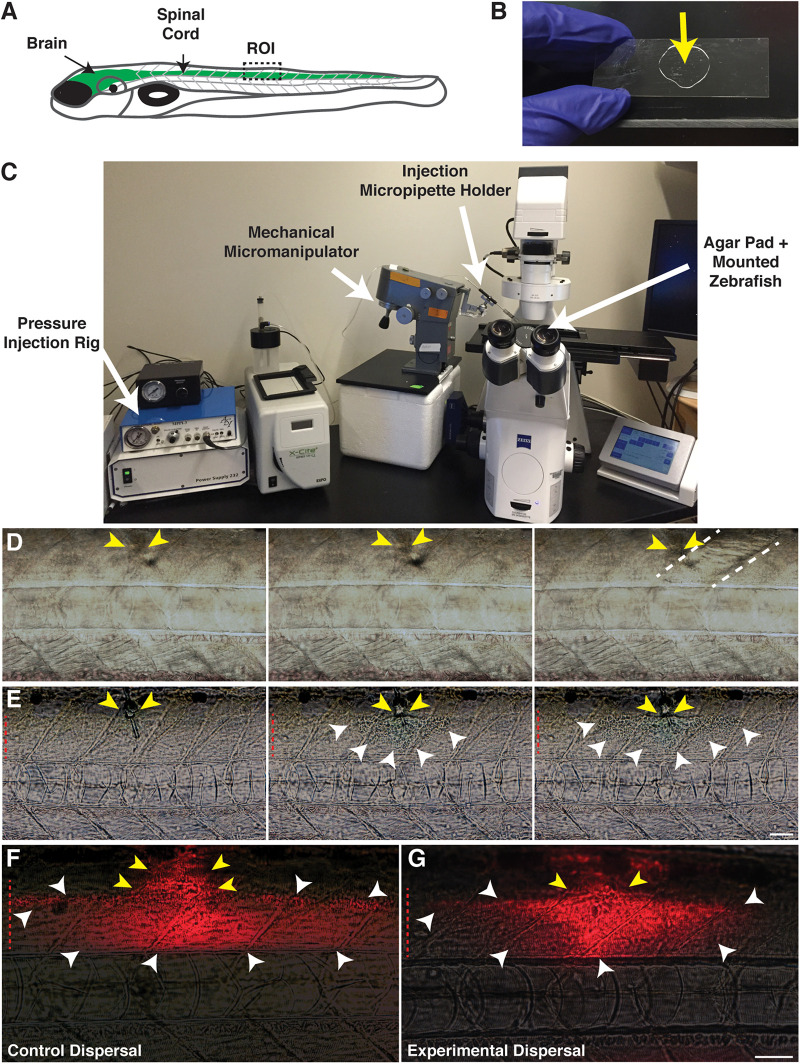
Focal delivery of solutions into the zebrafish spinal cord. **(A)** Cartoon of a zebrafish larvae identifying the brain and spinal cord (green). The boxed region identifies the injection site/targeted region of interest (ROI). **(B)** An agar pad (arrow) solidified on a borosilicate glass coverslip is used as a mounting platform for larvae to perform spinal cord injections. **(C)** An agar pad containing mounted larvae is placed on a microscope stage, equipped with a pressure injection rig and mechanical micromanipulator to hold the injection micropipette. **(D)** Muscle injections are identified by a wavy appearance following dispersal of the injection solution. Yellow arrows identify the injection site. Dotted lines denote the muscle segment injected. **(E)** Solutions remain localized within the spinal cord following a successful spinal cord injection. Yellow arrows identify the injection site in the dorsal spinal cord. White arrowheads identify the solution dispersal region. **(F)** Dispersal region of a control solution containing water and the fluorescent tracer, Dextran-647. Yellow arrows identify the injection site. White arrowheads identify the dispersal region. **(G)** Dispersal region of a lysolecithin solution including the fluorescent tracer Dextran-647. Yellow arrows identify the injection site. White arrowheads identify the dispersal region. Red dashed lines denote the spinal cord. Scale bar, 50 μm.

In order to inject into the spinal cord of 4–6 days post-fertilization (dpf) larvae (Figure 1A) to create focal areas of damage, we mounted larvae laterally on 2% agar pads solidified onto 22 mm × 60 mm borosilicate glass coverslips (Figure 1B). Coverslips containing mounted larvae were placed directly onto a Zeiss Axio Observer Z1 microscope stage equipped with an ASI MPPI-3 pressure injection rig and mechanical micromanipulator (Figure 1C). A micropipette needle containing either a 1× phosphate-buffered saline (PBS) control solution or a 0.875% lysolecithin experimental solution was inserted in the dorsal trunk and injected into the larvae *via* passive diffusion. When the micropipette was not inserted successfully into the spinal cord, the solution was inappropriately injected into the muscle, evidenced by the muscle appearing disturbed upon solution dispersal (Figure 1D). Successful injections resulted in the solution remaining localized within the spinal cord (Figure 1E). To confirm that injection volumes were comparable between the control and experimental groups, we included a fluorescent dye (Dextran-647) with the injection cocktail. In these injections, we observed comparable dispersal between the control (Figure 1F) and experimental solutions (Figure 1G), which on average, was 1–2 somites both anterior and posterior to the injection site. Additionally, we observed a similar survival rate 24 h post-injection for both groups, with the control survival at 72.67% (*n* = 236) and experimental survival at 69.4% (*n* = 183). From these studies, we present a targeted method to dispense solutions focally and precisely into the larval zebrafish spinal cord.

### Lysolecithin Alters the Number of Sox10^+^ Cells in the Zebrafish Spinal Cord

Myelination in the zebrafish CNS occurs in an anterior to posterior manner, commencing around 3 dpf (Almeida et al., 2011; Czopka et al., 2013). Therefore, the presence of maturing myelin sheaths between 4 and 6 dpf, coupled with the transparency of transgenic larvae, is ideal for *in vivo* imaging of the oligodendrocyte lineage cell (OLC) response following exposure to a demyelinating agent, such as lysolecithin. Previous publications have reported a decrease in oligodendrocyte number, demyelination, oligodendrocyte progenitor cell (OPC) proliferation, and OPC migration to the focal injury after injection of lysolecithin into the mammalian spinal cord (Blakemore et al., 1977; Kotter et al., 2001; Blakemore and Franklin, 2008; Lau et al., 2012; Münzel et al., 2014; Azin et al., 2015; Keough et al., 2015). Therefore, we sought to determine if lysolecithin similarly altered OLCs in our model.

**FIGURE 2:**
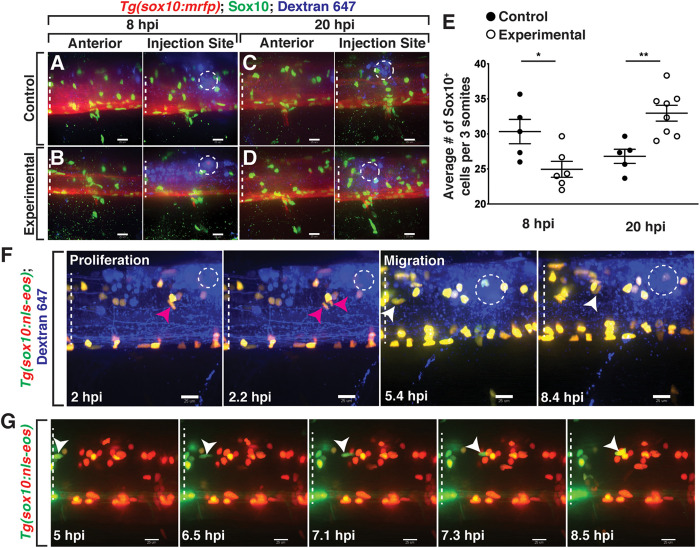
Lysolecithin alters the number and behavior of Sox10^+^ cells in the spinal cord. All images are lateral views of the spinal cord with anterior to the left and dorsal to the top. **(A–D)** 4 dpf *Tg*(*sox10:mrfp*) zebrafish larvae injected with either a control solution [control, **(A,C)**] or lysolecithin [experimental, **(B,D)**] containing the fluorescent tracer Dextran-647 to identify the injection site. Injected zebrafish were fixed at 8 hpi **(A,B)** or 20 hpi **(C,D)** and labeled with an antibody to Sox10 (green). **(E)** Quantification of the average number of Sox10^+^ cells within the injection site and spanning the width of approximately three motor nerves to capture the effects from lysolecithin (experimental) or control solutions. *p = 0.0252 at 8 hpi (*n* = 5 control; *n* = 6 experimental) and **p = 0.0033 at 20 hpi (*n* = 5 control; *n* = 8 experimental). Statistical significance was measured using an unpaired *t*-test. **(F)** Time-lapse imaging of proliferation (red arrowhead) and migration (white arrowhead) of *sox10*^+^ cells in a *Tg*(*sox10:nls-eos*) zebrafish larva following injection of lysolecithin at 4 dpf. White dashed ellipse denotes the injection site. **(G)** Time-lapse movie following injection of lysolecithin and photoconversion of the lesion area in a 4 dpf *Tg*(*sox10:nls-eos*) zebrafish larva. Red fluorescence denotes the photoconverted cells within the lesion. Green fluorescence denotes the cells outside of the lesion. Arrowhead identifies a *sox10*^+^ cell anterior to the injection site that migrates posteriorly into the drug dispersal region. White dashed lines denote the spinal cord. Scale bars, 25 μm.

To quantify the effects on OLC number in our model, we injected either a lysolecithin cocktail (0.875% lysolecithin + 0.21% Dextran-647 in PBS) or a control solution (PBS + 0.21% Dextran-647) into the spinal cord of 4 dpf *Tg*(*sox10:mrfp*) larvae, where *sox10* regulatory sequences drive the expression in OLCs. Following injection, we fixed larvae at distinct time points post-injection and performed whole mount immunohistochemistry with an antibody specific to zebrafish Sox10 to count the number of Sox10^+^ cells in the spinal cord (Binari et al., 2013). As a control, we quantified the number of Sox10^+^ cells anterior to the injection/dispersal region within a region of the spinal cord spanning approximately three motor nerves in width to avoid effects caused by the injection injury itself and observed no difference in Sox10^+^ cell number at 20 hpi (Figures 2A–D, left panels, control average, *n* = 5, 29.3 Sox10^+^ cells, experimental average, *n* = 6, 31.3 Sox10^+^ cells, *p* = 0.3391). We also quantified within the injection/dispersal region to capture the effects from either the lysolecithin or control solution (Figures 2A–E, right panels). The region posterior to the injection site was not quantified to reduce the risk of capturing a decrease in the number of OLCs that might be secondary to Wallerian degeneration (Waller, 1851). At 8 hours post-injection (hpi) within the injection site dispersal region, we observed a significant reduction in the number of Sox10^+^ cells (Figure 2E) in the lysolecithin-injected group (Figure 2B, right panel) (average 24.94, *n* = 6) as compared with control larvae (Figure 2A, right panel) (average 30.33, *n* = 5), demonstrating that lysolecithin is toxic to zebrafish OLCs. When we looked at 20 hpi, we observed an increase in the number of Sox10^+^ cells in lysolecithin-injected larvae (Figure 2D, right panel) (average 32.96, *n* = 8) as compared with control-injected larvae (Figure 2C, right panel) (average 26.8, *n* = 5), which we hypothesize is due to OPC proliferation, a phenomena reported in mammalian lysolecithin-induced demyelination models (Nait-Oumesmar et al., 1999; Keough et al., 2015; Sahel et al., 2015).

To determine if OLCs were proliferating after exposure to lysolecithin and to investigate whether migration also occurred in our model, we injected the lysolecithin cocktail into 4–6 dpf *Tg*(*sox10:nls-eos*) (McGraw et al., 2012; Prendergast et al., 2012) larvae and used *in vivo*, time-lapse imaging to follow *sox10*^+^ cells with an imaging interval of 8 min. Following injection of lysolecithin, we observed that *sox10*^+^ OLCs near the injection site divide (Figure 2F and Supplementary Movie 1). We also observed that OLCs migrate into the injection lesion from outside of the dispersal region (Figure 2F and Supplementary Movie 1). To investigate if *sox10*^+^ cells also migrated from areas significantly anterior to the lesion, we used *Tg*(*sox10:nls-eos*) embryos to selectively label OLCs within a two-somite region encompassing the dispersal region. Following injection of lysolecithin at 4 dpf, we photoconverted the Eos protein, changing the green fluorescence to red *via* UV-light-induced photoconversion (Wiedenmann et al., 2004; Stys et al., 2012). We then used *in vivo*, time-lapse imaging to track the migration of both green (non-photoconverted) and red (photoconverted) OLCs with an imaging interval of 10 min (Figure 2G). Starting approximately 6.5 hpi, we observed that green *sox10*^+^ OLCs from anterior to the injection dispersal region migrate posteriorly into the lesion (Figure 2G). Interestingly, we rarely observed that OLCs within the lysolecithin dispersal region migrate during our imaging. This data demonstrates that a subset of *sox10*^+^ OLCs found in the lesion originate from areas anterior to the drug dispersal region. Taken together, these results demonstrate that lysolecithin affects *sox10*^+^ OLCs in the zebrafish spinal cord.

### Lysolecithin Induces Myelin Membrane Changes in Zebrafish Larvae

**FIGURE 3:**
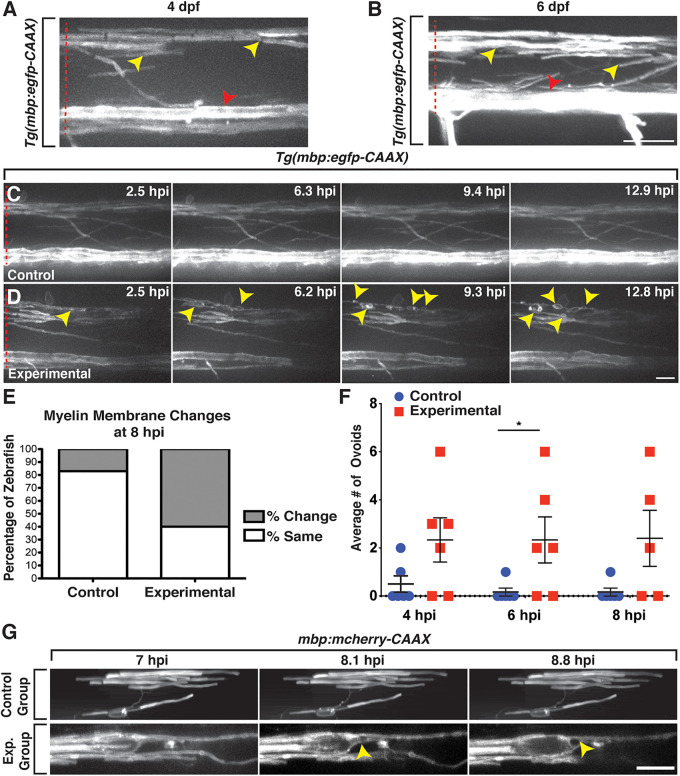
*mbp*^+^ membrane changes are observed following exposure to lysolecithin. All images are lateral views of the spinal cord with anterior to the left and dorsal to the top. **(A,B)** Expression of *mbp:egfp-CAAX* appears as sheath-like structures in the dorsal and ventral spinal cords in 4 **(A)** and 6 dpf **(B)** zebrafish larvae. **(C)** Following injection of a control solution into the spinal cord of a 4 dpf *mbp:eegfp-CAAX* larva, the *mbp*^+^ membrane sheaths remain unchanged throughout the duration of the time-lapse movie. **(D)** Following injection of a lysolecithin (experimental) solution into the spinal cord of a 4 dpf *mbp:egfp-CAAX* larva, the *mbp*^+^ sheaths dynamically change to form ovoids (arrowheads). **(E)** Quantification of the percentage of zebrafish with *mbp:egfp-CAAX* membrane changes at 8 hpi. *p* < 0.0001; *n* = 6 control; *n* = 5 experimental. **(F)** Quantification of the average number of *mbp:egfp-CAAX* ovoids observed at 4, 6, and 8 hpi demonstrates an increase in the number of ovoids within the lysolecithin (experimental) group (*n* = 6) when compared with the control group (*n* = 6). At 6 hpi, *p = 0.0493. **(G)** Mosaically labeled oligodendrocytes by injection of *mbp:mcherry-CAAX.* Larvae injected with a control solution (control, top) or lysolecithin (exp. group, bottom) at 6 dpf. Following injection of a control solution (top), the oligodendrocyte remains relatively unchanged throughout the course of the time-lapse movie. In contrast, an ovoid-like structure is observed within the *mbp:mcherry-CAAX*^+^ oligodendrocyte beginning around 8.1 hpi. Red dashed lines denote the spinal cord. Scale bars, 25 μm.

Because we observed a reduction in Sox10^+^ OLC number and subsequent OPC proliferation and recruitment in lysolecithin-injected larvae, we next sought to investigate whether lysolecithin-induced myelin membrane changes by performing *in vivo*, time-lapse imaging in *Tg*(*mbp:egfp-CAAX*) (Almeida et al., 2011) larvae, where *myelin basic protein* (*mbp*) is labeled with membrane-tethered GFP. *mbp* is expressed in anterior regions of the zebrafish CNS beginning around 60 hpf (Almeida et al., 2011). At later stages, between 4 and 6 dpf, we observed stable GFP^+^ membrane sheaths in both the ventral and dorsal spinal cords (Czopka and Lyons, 2011; Kearns et al., 2015; Figures 3A,B). Following injection of the PBS control solution into 4 dpf *Tg*(*mbp:egfp-CAAX*) larvae, we observed minimal to no changes to the GFP^+^ membrane near the injection site throughout the duration of our 16-h time-lapses with an imaging interval of 6 min (*n* = 6) (Figure 3C). In contrast, we observed distinct changes in GFP^+^ myelin membrane near the lesion after injection of the lysolecithin solution into the spinal cord of 4 dpf *Tg*(*mbp:egfp-CAAX*) larvae (*n* = 6) (Figure 3D). Specifically, we imaged myelin one somite anterior to the injection site so as to avoid possible damage induced by the injection itself. In these time-lapses, we observed that tight GFP^+^ membrane sheaths change after lysolecithin injection to take on a more ovoid-like appearance, beginning around 2.5 hpi (Figure 3D). Formation of the ovoid-like/onion bulb structures has previously been reported as evidence of myelin degradation (Acar et al., 2004), and we did not observe the same phenotype in control-injected larvae (Figure 3C).

Because the *mbp*^+^ membrane ovoids were obvious by 8 hpi in lysolecithin-injected larvae, we decided to quantify the percentage of zebrafish demonstrating GFP^+^ membrane changes within the dorsal spinal cord at this time point and observed a significant difference between the control and experimental groups, with approximately 17% of control zebrafish (*n* = 6) with GFP^+^ membrane changes within the dispersal area as compared with 60% of zebrafish injected with lysolecithin at 8 hpi (*n* = 5; Figure 3E). Additionally, we quantified the number of myelin ovoid structures within the control and experimental dispersal regions from 4 to 8 hpi, time points where the GFP^+^ membrane was dynamically changing in lysolecithin-injected larvae. In the lysolecithin-injected larvae, we observed an increase in the average number of GFP^+^ membrane ovoids that formed (*n* = 6 for both groups, at 6 hpi *p* = 0.0493, Figure 3F).

Because *mbp:egfp-CAAX* is a stable transgenic line, it is difficult to visualize individual oligodendrocyte membrane changes. Therefore, to investigate individual *mbp*^+^ membrane sheath dynamics, we injected a plasmid for *mbp:mcherry-CAAX* (Mensch et al., 2015) into one-cell embryos to mosaically label *mbp*^+^ oligodendrocytes in the spinal cord. At 6 dpf, we then injected either a PBS control solution or lysolecithin posterior to an *mbp*^+^ oligodendrocyte, such that the oligodendrocyte would reside anterior to the injection site but would be exposed to the injected solutions (Figure 3G). Using *in vivo* imaging with an image acquisition interval of 10 min, we observed an *mbp*^+^ ovoid form at approximately 8.1 hpi within the lysolecithin-injected group (*n* = 3; Figure 3G, bottom), but this phenotype was not observed in the control-injected larvae (*n* = 2; Figure 3G, top). Taken together, our results demonstrate that lysolecithin induces *mbp*^+^ oligodendrocyte membrane changes in the zebrafish spinal cord.

### Axons Are Indistinguishable Between Lysolecithin- and Control-Injected Larvae

**FIGURE 4:**
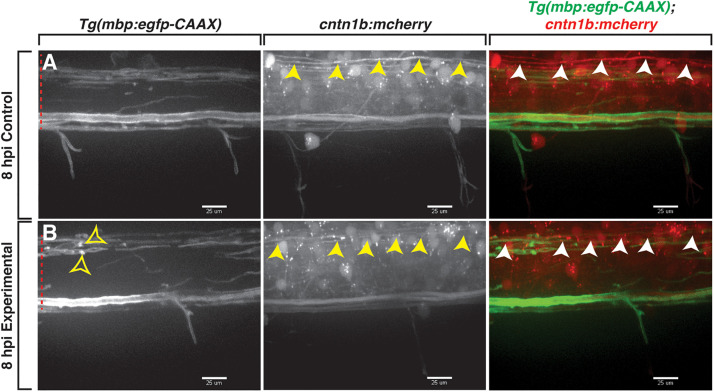
Axons are indistinguishable between control and lysolecithin-injected larvae. All images are lateral views of the spinal cord with anterior to the left and dorsal to the top. **(A,B)** mCherry^+^ axons (arrowheads) within the control **(A)** and lysolecithin (experimental) **(B)** dispersal regions are indistinguishable at 8 hpi. *mbp*^+^ ovoids are denoted with open arrowheads. Red dashed lines denote the spinal cord. Scale bars, 25 μm.

In mammals, previous studies demonstrate that lysolecithin causes minimal damage to axons (Blakemore et al., 1977; Keough et al., 2015). To investigate the effects of lysolecithin on axons in our focal injection model, we injected either a control solution or lysolecithin into the spinal cord of 4 dpf *Tg(mbp:egfp-CAAX);Tg(cntn1b:mcherry)* (Almeida et al., 2011; Czopka et al., 2013) larvae to label myelin with membrane-tethered GFP and axons with cytosolic mCherry. This transgenic line labels axons that have previously been described as being preferentially myelinated early in development in the zebrafish spinal cord (Czopka et al., 2013), and therefore, it allows us to specifically look at axonal integrity in relation to the lysolecithin-induced demyelination we observe. Using *in vivo* imaging, we observed that axons were indistinguishable between the control (*n* = 3; Figure 4A) and the lysolecithin-injected larval spinal cords (*n* = 2; Figure 4B) at 8 hpi, a time point in which we previously observed active *mbp*^+^ myelin changes *via in vivo*, time-lapse imaging (Figure 3D). From these data, we conclude that the effects observed on *mbp*^+^ membrane sheaths within the lysolecithin group described above were not secondary to axonal degeneration.

### Professional Phagocytes Are Recruited to Lysolecithin-Induced Lesions

Macrophages and microglia respond to 1% lysolecithin injections in mammalian models and upon application to the adult zebrafish optic nerve and injection into the zebrafish spinal cord (Kotter et al., 2001; Münzel et al., 2014; Cunha et al., 2020). Therefore, we evaluated if macrophages and microglia were recruited to the focal lesion in our lysolecithin injection model in the larval zebrafish spinal cord.

**FIGURE 5:**
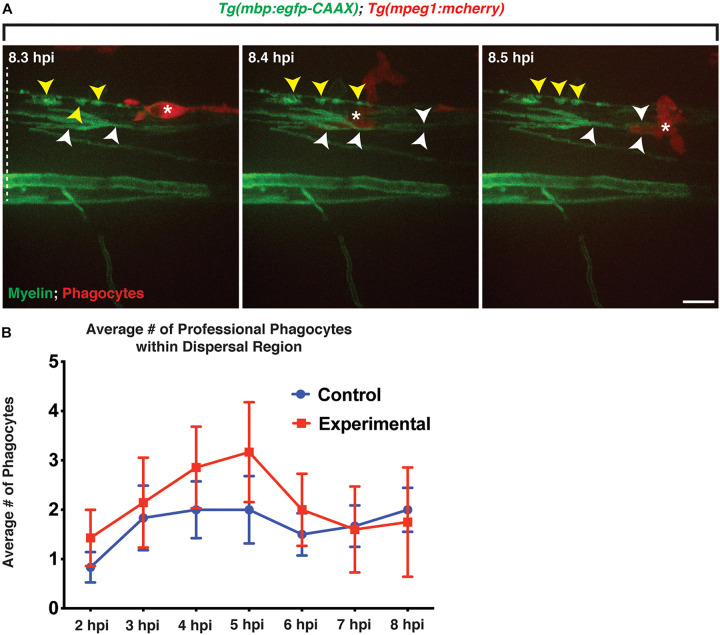
Professional phagocytes traverse through *mbp*^+^ membrane layers. All images are lateral views of the spinal cord with anterior to the left and dorsal to the top. **(A)** Following injection of lysolecithin, a mCherry^+^ phagocyte is observed traversing through *mbp*^+^ membrane layers within the lesion area, physically moving the layers during its migration. White arrowheads identify the layers that are moved by the phagocyte, and yellow arrowheads denote the myelin ovoids. **(B)** Quantification of the average number of professional phagocytes responding to the lesion following injection of a control (control) solution (*n* = 6) or lysolecithin (experimental) (*n* = 7); *p* = 0.0864. White dashed line denotes the spinal cord. Scale bars, 25 μm.

To investigate professional phagocyte recruitment in our model, we injected either a control solution or lysolecithin into the spinal cord of 4 and 6 dpf *Tg(mbp:egfp-CAAX);Tg(mpeg1:mcherry)* (Almeida et al., 2011; Ellett et al., 2011) larvae, where *mbp*^+^ oligodendrocytes are labeled with membrane GFP, and macrophages/microglia were labeled with cytosolic mCherry. In both 4 and 6 dpf larvae, we observed that *mpeg*^+^ cells move quickly throughout the CNS, maneuvering through *mbp*^+^ myelin membrane layers. In an *in vivo*, time-lapse movie with an imaging interval of 6 min of a 4 dpf larva injected with lysolecithin, we observed that maneuvering mCherry^+^ phagocytes physically displaces *mbp*^+^ membrane sheaths while traversing through the dispersal region within the spinal cord (Figure 5A and Supplementary Movie 2).

We next quantified the number of *mpeg*^+^ phagocytes present in the lesion area to determine if larvae injected with lysolecithin had a greater response by professional phagocytes. Following injection of a control solution or lysolecithin into the spinal cord of 4 dpf *Tg(mbp:egfp-CAAX);Tg(mpeg1:mcherry)* larvae, we saw a modest increase in *mpeg*^+^ phagocytes within the lysolecithin-injected group from 2 to 6 hpi as compared with control injections (*n* = 6 control; *n* = 7 experimental; Figure 5B). Taken together, these results are consistent with the possibility that *mpeg*^+^ phagocytes are recruited to the lesion area, and this recapitulates the macrophage/microglia recruitment response that has been reported in mammalian lysolecithin models (Kotter et al., 2001; Münzel et al., 2014).

**FIGURE 6:**
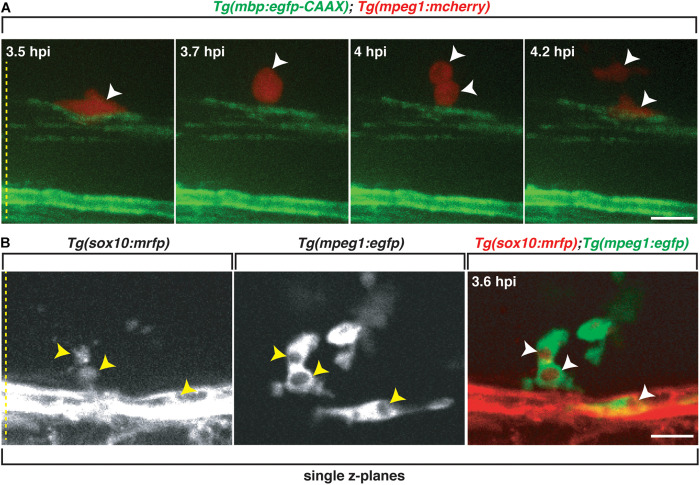
Professional phagocytes proliferate and clear Sox10^+^ debris. All images are lateral views of the spinal cord with anterior to the left and dorsal to the top. **(A)** Following injection of lysolecithin into a 4 dpf zebrafish spinal cord, a mCherry^+^ phagocyte recruited to the lysolecithin lesion proliferates (arrowheads). **(B)** Following injection of lysolecithin into a 6 dpf zebrafish spinal cord, *sox10*^+^ debris is cleared by an *mpeg*^+^ cell. Arrowheads identify the *sox10*^+^ debris within *mpeg*^+^ vacuoles in single z-planes. Yellow dashed lines denote the spinal cord. Scale bars, 25 μm.

In addition to phagocyte migration toward the lesion, macrophages and microglia proliferate during demyelination and inflammation (Chiang et al., 1996; Remington et al., 2007; Jenkins et al., 2011). To investigate if this occurs in our lysolecithin model, we injected lysolecithin into the spinal cord of 4 dpf *Tg(mbp:egfp-CAAX);Tg(mpeg1:mcherry)* larvae and performed *in vivo*, time-lapse imaging with an imaging interval of 15 min. In these movies, we captured *mpeg*^+^ cells proliferating within the lysolecithin dispersal region (Figure 6A and Supplementary Movie 3), demonstrating that professional phagocytes migrate into the lesion and proliferate in response to the lysolecithin insult.

Because we observed that *mbp*^+^ membrane changes within lysolecithin-injected larvae, we next wanted to determine if professional phagocytes were responding to clear oligodendrocyte/myelin debris. To capture the clearance of oligodendrocyte debris *in vivo*, we injected lysolecithin into 6 dpf *Tg*(*sox10:mrfp; mpeg1:egfp*) larvae and performed *in vivo*, time-lapse imaging with an imaging interval of 10 min. As described above, *mpeg*^+^ cells were recruited to the lysolecithin dispersal region. By 3.6 hpi, we observed *mpeg1*^+^ cells with distinct cytoplasmic vacuoles surrounding *sox10*^+^ debris (Figure 6B and Supplementary Movie 4). Taken together, our results demonstrate that our focal lysolecithin injection model in zebrafish recapitulates the professional phagocyte response previously reported in lysolecithin-injected mammalian models (Kotter et al., 2001; Kucharova et al., 2011; Rawji and Yong, 2013; Doring et al., 2015).

### Oligodendrocyte Cytoskeleton Dynamics Following Exposure to Lysolecithin

Actin dynamics are essential for myelin sheath formation (Nawaz et al., 2015; Samanta and Salzer, 2015; Zuchero et al., 2015). Specifically, filamentous actin (F-actin) is integral for initiating myelin wrapping and, upon completion of myelination, is disassembled from the inner tongue/leading edge and becomes localized to the lateral edges of the myelin sheath (Nawaz et al., 2015; Samanta and Salzer, 2015; Zuchero et al., 2015). Interestingly, actin polymerization is involved in peripheral myelin fragmentation and ovoid/onion bulb formation following injury *in vitro*, and inhibiting actin polymerization prevents myelin ovoid formation (Jung et al., 2011). Furthermore, cytoskeletal plasticity was recently identified as an important component of demyelination, suggesting that mechanisms within the oligodendrocyte cytoskeleton may be actively involved in reacting to demyelinating insults (Locatelli et al., 2015).

**FIGURE 7:**
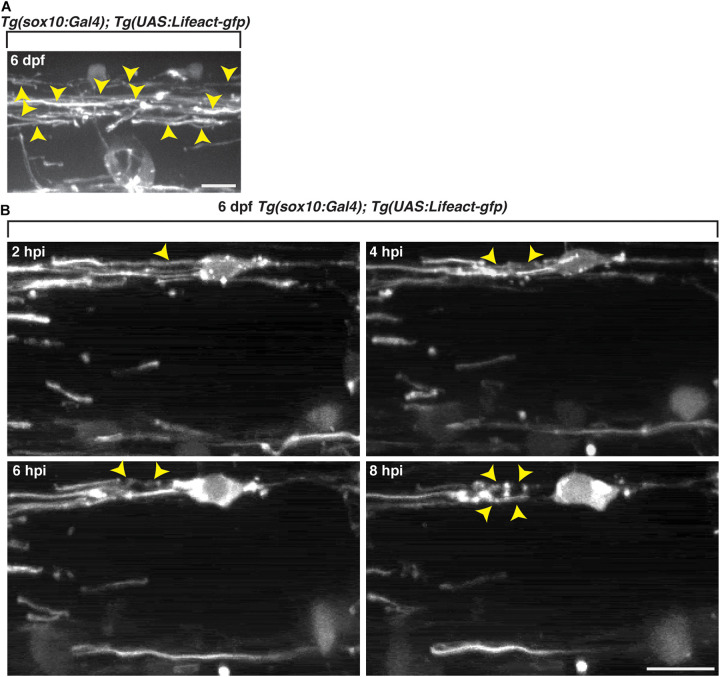
Oligodendrocyte cytoskeletal components dynamically change following exposure to lysolecithin. All images are lateral views of the spinal cord with anterior to the left and dorsal to the top. **(A)** *In vivo* imaging of *Tg*(*sox10:Gal4; UAS:Lifeact-gfp*) larvae at 6 dpf. Arrowheads denote the actin in sheath formation. **(B)** Following injection of lysolecithin into a 6 dpf *Tg*(*sox10:Gal4; UAS-Lifeact-gfp*) zebrafish, *in vivo* time-lapse imaging reveals that GFP^+^ F-actin dynamically changes from a sheath-like arrangement (2 hpi) to forming ovoid-like structures (8 hpi). Arrowheads identify the GFP^+^ F-actin changes in a *sox10*^+^ cell. Scale bars, 25 μm.

To investigate actin dynamics within *sox10*^+^ oligodendrocytes *in vivo*, we imaged with an interval of 10 min, control 6 dpf *Tg(sox10:Gal4);Tg(UAS:Lifeact-gfp)* larvae (Helker et al., 2013) and observed GFP^+^ F-actin arranged as sheath-like structures (Figure 7A), reminiscent of the appearance of *mbp*^+^ myelin membrane sheaths (Figure 3). To evaluate changes in F-actin, we injected lysolecithin into 6 dpf *Tg(sox10:Gal4);Tg(UAS:Lifeact-gfp)* larvae, a time point when the GFP^+^ sheath-like structures were stable in control-injected larvae. At 2 hpi, GFP^+^ F-actin within *sox10*^+^ cells appeared sheath-like. However, by 4 hpi, we observed GFP^+^ F-actin rearranging from a sheath-like structure to form ovoid-like structures by 8 hpi (Figure 7B). The arrangement of the F-actin from sheath-like into ovoid-like structures is reminiscent of the *mbp*^+^ membrane changes described above and occurred at a similar time point post-injection. These results suggest that the dynamic responses from components of the oligodendrocyte cytoskeleton appear to be involved in the lysolecithin-induced membrane changes that we observed post-injection.

## Discussion

### Toxin-Induced Animal Models for Recapitulating CNS Damage

The use of toxin-induced demyelination models has been imperative for investigating mechanics underlying oligodendrogliopathies as well as processes involved in remyelination (Blakemore et al., 1977; Foote and Blakemore, 2005; Blakemore and Franklin, 2008; Ransohoff, 2012). Although toxin-induced demyelination models lack an ongoing immune response, there is a unique advantage of being able to separate demyelination events from processes underlying remyelination for studying neurodegenerative disorders, such as MS (Miller and Fyffe-Maricich, 2010; Ransohoff, 2012). Because of this spatiotemporal predictableness, toxin-induced demyelination models can also be useful for screening therapeutic strategies that promote remyelination (Ransohoff, 2012).

Mammalian models commonly used to study MS, including EAE and toxin-induced demyelination models, as well as clinical samples, are not amenable for investigating mechanics and processes underlying myelin destruction and subsequent repair *in vivo*. By developing a focal lysolecithin injection model in zebrafish, we can visualize *mbp*^+^ membrane changes in a live, intact vertebrate model system and investigate mechanics and cellular interactions involved in myelin breakdown and repair. Recently, a paper using injection of lysolecithin into larval zebrafish trunks also described demyelination, and their data is similar to ours (Cunha et al., 2020); in the future, we envision that these focal injection models could be used as a first-pass drug-screening tool to investigate the safety and efficacy of novel compounds that either inhibit lysolecithin-induced damage, or promote its repair, to potentially reveal therapeutic strategies for treating demyelinating disorders, such as MS.

### Observing *mbp*^+^ Membrane Changes *in vivo*

Intramyelinic vacuoles and fluid accumulation within the myelin sheath have been reported following lysolecithin-induced demyelination (Triarhou and Herndon, 1985) and in other toxin-induced demyelination models (Blakemore, 1973, 1974). In our focal lysolecithin injection model, we were able to visualize *mbp*^+^ membrane changes and observed that these sheaths change to form ovoid-like vacuoles *in vivo*, in real time. However, future studies are needed to confirm that the ovoids we see in our *in vivo* imaging assays are from intramyelinic edema (Hirano et al., 1965; Hirano and Llena, 2006; Kaufmann et al., 2012).

Additionally, the dynamic rearrangement of F-actin within the oligodendrocyte cytoskeleton suggests that actin polymerization may be involved with the formation of the *mbp*^+^ ovoids that we observed in our *in vivo*, time-lapse imaging. Actin dynamics are essential for myelin sheath formation, and it is reasonable to assume that they play an integral role in kinetics underlying myelin membrane breakdown and repair following a toxin-induced insult. Additionally, after *in vitro* peripheral nerve injuries, actin polymerization is involved in the formation of onion bulbs, and inhibiting actin polymerization prevents their formation (Jung et al., 2011), demonstrating that actin polymerization is actively involved in myelin membrane sheath changes. Mechanisms within the oligodendrocyte cytoskeleton may also be actively involved in reacting to insults as β-actin and β-tubulin gene transcription are upregulated in response to diphtheria-toxin oligodendrocyte death and EAE autoimmune inflammation (Locatelli et al., 2015). To our knowledge, there have been no reports on actin dynamics following lysolecithin-induced damage. Thus, future studies are needed in our model of demyelination to determine if actin dynamics are actively rearranging the myelin sheath to form these ovoid-like structures, or are simply a passive bystander that dynamically rearranges as a result of the myelin sheath changes following lysolecithin-induced damage.

### Professional Phagocyte Response Following Lysolecithin Insult

A previous report demonstrates a rapid recruitment of macrophages and microglial cells following injection of lysolecithin into the mouse spinal cord (Ousman and David, 2000). In the study by Ousman and David (2000), phagocytes were observed within the injection region by 6 hpi, and ultrastructural analysis at 4 dpi revealed macrophages containing myelin debris. Microglia/macrophages have also been shown to proliferate extensively in early demyelination (Matsumoto et al., 1992; Schönrock et al., 1998; Raivich and Banati, 2004) as well as in the EAE model (Rinner et al., 1995) and lysolecithin-induced demyelination in primates (Dousset et al., 1995). In the adult zebrafish optic nerve as well as the larval spinal cord, there is an increase in the number of professional phagocytes (Münzel et al., 2014; Cunha et al., 2020).

In our model, we also observe that *mpeg1*^+^ professional phagocytes respond to the injection region rapidly. The *mpeg1*^+^ professional phagocytes migrated to the lesion and proliferated, as previously reported in a primate model of lysolecithin-induced demyelination (Dousset et al., 1995). Since lysolecithin acts as a chemotactic factor for peripheral macrophages (Quinn et al., 1988), we expected to see an increase in the number of phagocytes within the lysolecithin lesion. Although there was an increase in the average number of professional phagocytes that we observed from 2 to 6 hpi, the results were not statistically significant between the control and lysolecithin-injected larvae, likely because the control larvae also experienced an injury from the injection site.

Phagocytes have been described as having both beneficial and detrimental roles in MS (Huitinga et al., 1990; Bitsch et al., 2000; Hinks and Franklin, 2000; Ousman and David, 2000; Arnett et al., 2001; Copelman et al., 2001; Kotter et al., 2001, 2005; Lassmann et al., 2001; Mason et al., 2001; Hill et al., 2004; Rose et al., 2004; Lampron et al., 2015; Domingues et al., 2016). Zebrafish offer a unique advantage for investigating the transition from beneficial phagocytic responses to detrimental behaviors *in vivo* as macrophages can be depleted using a nitroreductase genetic model (Pisharath et al., 2007; Curado et al., 2008; Pisharath and Parsons, 2009), allowing for inducible and reversible depletion of *mpeg1*^+^ cells. *Escherichia coli* nitroreductase can be expressed under the control of the *mpeg1* promoter (Palha et al., 2013; Travnickova et al., 2015) by crossing *Tg*(*mpeg1:Gal4FF*)*^*gl25*^* (Ellett et al., 2011) and *Tg*(*UAS:nfsB-mCherry*)*^*c26*^* (Davison et al., 2007) lines together. To initiate cell depletion, larvae are immersed in the prodrug metronidazole, and nitroreductase converts the metronidazole into a cytotoxin, resulting in cell death. The effects are reversible upon removing the zebrafish from the prodrug solution. This genetic ablation model allows for spatial and temporal control over cell ablation and can be used in our focal lysolecithin injection model to investigate the impact that the absence of *mpeg1*^+^ cells has on de- and remyelination events in real time, in a living vertebrate organism, feat mammalian systems currently cannot offer. These types of experiments will provide us with a better understanding about the roles and temporal dynamics of professional phagocytes in lysolecithin-induced injury and repair.

In conclusion, the lysolecithin injection model we developed using zebrafish larvae recapitulates phenotypes that have been described in mammalian systems following injection of lysolecithin, with a major advantage of being able to visualize damage in real time, *in vivo.* An important difference is that our study uses larvae and most mammalian studies utilize adults. Future studies investigating the temporal dynamics of juvenile mammalian demyelination would be interesting given the rapid responses we see in our model. Finally, this focal injection model may become a useful drug-screening assay for revealing therapeutic strategies that help to prevent damage in myelinating glia or accelerate and enhance repair kinetics following damage.

## Experimental Procedures

### Fish Husbandry

Animal studies were approved by the University of Virginia Institutional Animal Care and Use Committee. Zebrafish strains used in this study included *Tg*(*mbp:egfp-CAAX*) (Almeida et al., 2011), *Tg[sox10*(*7.2*)*:mrfp]^*vu234*^* (Kucenas et al., 2008), *Tg*(*cntn1b:mCherry*) (Czopka et al., 2013), *Tg*(*mpeg1:mCherry*) (Ellett et al., 2011), *Tg*(*mpeg1:egfp*) (Ellett et al., 2011), *Tg*(*sox10:nls-eos*) (McGraw et al., 2012), *Tg*(*sox10:Gal4-vp16*) (Chung et al., 2013), and *Tg*(*UAS:Lifeact-GFP*)*^*mu271*^* (Helker et al., 2013). Table 1 identifies the expression of all lines used in this study. Embryos were produced by pairwise matings, raised at 28.5°C in egg water, staged according to hpf or dpf, respectively, and embryos of either sex were used for all experiments (Kimmel et al., 1995). Embryos used for lysolecithin injections, immunohistochemistry, and microscopy were treated with 0.003% phenylthiourea (PTU) in egg water to inhibit pigmentation.

**TABLE 1:** Descriptions and abbreviations of transgenic lines used in this study.

Transgene name	Description of expression
*Tg*(*mbp:egfp-CAAX*)	Membrane eGFP in *mbp*^+^ cells (oligodendrocytes)
*Tg*(*mpeg1:mcherry*)	Cytosolic mCherry in *mpeg*^+^ cells (macrophages, microglia)
*Tg*(*mpeg1:egfp*)	Cytosolic eGFP in *mpeg*^+^ cells (macrophages, microglia)
*Tg*(*sox10:nls-eos*)*^*w18*^*	Nuclear localized Eos in *sox10*^+^ cells (OPCs, oligodendrocytes)
*Tg*(*sox10:mrfp*)*^*vu234*^*	Membrane RFP in *sox10*^+^ cells (OPCs, oligodendrocytes)
*Tg*(*sox10:Gal4*)	Gal4 specifically expressed in *sox10*^+^ cells (OPCs, oligodendrocytes)
*Tg*(*UAS:Lifeact-gfp*)*^*mu271*^*	UAS promoter driving Lifeact-GFP to label filamentous actin (F-actin)
*Tg*(*cntn1b:mcherry*)	Cytosolic mCherry in mature neurons and axons

*All lines were stable, germline transgenics. Only cell types pertinent to this study are listed for each transgene.*

### Individual Oligodendrocyte Labeling

To mosaically label oligodendrocytes, one-cell embryos were injected with 12.5 ng/μl of a DNA plasmid encoding for *mbp:mcherry-CAAX* (Mensch et al., 2015) (courtesy of Lyons) and 25 ng/μl transposase RNA.

### Spinal Cord Injections

Lysolecithin (1-palmitoyl-Sn-glycero-3-phosphocholine, Sigma L5254-25mg) was dissolved in saline at a 1% stock concentration and stored at −20°C. Thin wall glass microcapillaries (World Precision Instruments, Inc., glass thin wall with filament 1.0 mm OD, four in lengths, Item No. TW100F-4) were pulled for microinjections using a micropipette puller (Sutter Instrument Co., Flaming/Brown Micropipette Puller, Model P-97), and the capillary tip was opened using forceps (Student Dumont #5 Inox Forceps, Fine Science Tools, Item #91150-20). For experimental injections, lysolecithin was diluted to 0.875% concentration in saline and loaded into the glass microcapillary using a Microloader^TM^ tip (Eppendorf Catalog No. 5242956003). For control injections, water or saline was loaded into the glass microcapillary using a Microloader^TM^ tip. Larvae 4–6 dpf were anesthetized using 3-aminobenzoic acid ester (Tricaine) and mounted laterally onto 2% agar pads solidified onto a borosilicate glass coverslip (22 mm × 60 mm; Fisherbrand Cover Glasses: Rectangles, Catalog No. 12-545-J). The coverslip with mounted larvae was placed onto a Zeiss Axio Observer Z1 microscope stage equipped with an ASI MPPI-3 pressure injection rig and mechanical micromanipulator. The micropipette needle, containing either the control or lysolecithin solution, was inserted into the larvae, starting from the dorsal spinal cord and moving ventrally within the spinal cord, between somites 16 and 18. Solutions were dispersed passively into the spinal cord at a pressure range below 16 psi, until the dispersal region spanned the width of one to two somites. Following injections, the glass coverslip containing the mounted larvae was removed from the microscope stage and placed into a Petri dish (Falcon Sterile Petri Dish, 100 mm × 15 mm style, REF 351029) with egg water. The coverslip and larvae were completely submersed in egg water until the agar pad was saturated, resulting in the larvae floating off the agar pad. A pipette pump (Bel-Art, Scienceware Pipette Pump 10 ml Pipettor, Cat. No. 378980000) with a 2 ml glass Pasteur pipet tip (VWR Pasteur Pipet Disposable BBD Borosilicate Glass, 5 3/4 inch size, Cat. No. 53283-916) was used to transfer the larvae into a separate Petri dish with egg water to allow the larvae to recover.

### *In vivo* Imaging

All embryos used for live imaging were transferred to egg water containing PTU at 24 hpf to inhibit pigment formation. At specified stages, embryos and larvae were anesthetized using 3-aminobenzoic acid ester (Tricaine), immersed in 0.8% low-melting point agarose, and mounted on their sides in glass-bottomed 35 mm Petri dishes (Electron Microscopy Sciences). Images were captured using either a 40× (numerical aperture 1.2) or a 63× (numerical aperture 1.2) water-immersion objective mounted on a motorized Zeiss Axio Observer Z1 microscope equipped with a Quorum WaveFX-X1 spinning disc confocal system (Quorum Technologies). For time-lapse imaging, *z*-stacks were collected at specified intervals, and three-dimensional datasets were compiled using MPEG-4 video compression at 10 frames per second (fps) and exported to QuickTime (Apple) to create movies. Image adjustments were limited to contrast enhancement and level settings using MetaMorph software, Adobe Photoshop, and ImageJ.

### Whole Mount Immunohistochemistry

Larvae were fixed in AB Fix [4% paraformaldehyde (PFA), 0.1% Triton-X 100, 1× PBS] for either 3 h at 23°C or overnight at 4°C, followed by a 5-min wash with PBSTx (1% Triton X-100, 1× PBS), a 5-min wash with DWTx (1% Triton X-100 in distilled water), a 5-min wash with acetone at 23°C, a 10-min wash with acetone at −20°C, and three 5-min washes with PBSTx. Larvae were preblocked in 5% goat serum/PBSTx for at least 1 h and incubated in primary antibody for 1 h at 23°C and overnight at 4°C. The primary antibody used in this study was a rabbit antibody to Sox10 (1:5,000) (Binari et al., 2013). Larvae were washed extensively with 1× PBSTx and stored in 50% glycerol–PBS at 4°C until imaging. Larvae were mounted on their sides in 0.8% low-melting point agarose on glass-bottomed 35 mm Petri dishes and imaged using the confocal microscope described above. Image adjustments were limited to contrast enhancement and level settings using MetaMorph software, Adobe Photoshop, and ImageJ.

### Data Quantification and Statistical Analysis

All graphically presented data represent the mean of the analyzed data. Statistical analyses and graphing were performed with GraphPad Prism software. The level of significance was determined by using an unpaired *t*-test or a chi-square test using a confidence interval of 95%. The data in plots and the text are presented as mean ± SEM.

## Data Availability Statement

The original contributions presented in the study are included in the article/Supplementary Material, further inquiries can be directed to the corresponding author/s.

## Ethics Statement

The animal study was reviewed and approved by the University of Virginia Institutional Animal Care and Use Committee.

## Author Contributions

AM conducted and analyzed all of the experiments. Both authors conceived the study and wrote the manuscript.

## Conflict of Interest

The authors declare that the research was conducted in the absence of any commercial or financial relationships that could be construed as a potential conflict of interest.

## Supplementary Material

Supplementary Movie 1 Sox10^+^ cells proliferate and migrate after injection of lysolecithin into the larval spinal cord. Panels are partially overlapping regions of the spinal cord in lysolecithin-injected (blue region) 4 dpf *Tg*(*sox10:nls-eos*) larvae imaged from 2 to 10 hpi. The right edge of the left panel is the left edge of the right panel. OLC proliferation is denoted by pink arrowheads (left panel), and a migratory OLC is denoted by a green dot (both panels). Images were taken every 8 min, and the movie runs at 10 fps.

Supplementary Movie 2 *mpeg*^+^ phagocytes migrate through myelin layers in lysolecithin-injected spinal cords. Lysolecithin was injected into 4 dpf *Tg(mbp:egfp-CAAX);Tg(mpeg1:mcherry)* larvae imaged from 2 to 10 hpi. Yellow box denotes the site of injection. Pink arrowheads denote the *mpeg*^+^ cells moving through myelin layers. Images were taken every 6 min, and the movie runs at 10 fps.

Supplementary Movie 3 *mpeg*^+^ phagocytes proliferate in lysolecithin-injected spinal cords. Lysolecithin was injected into 4 dpf *Tg(mbp:egfp-CAAX);Tg(mpeg1:mcherry)* larvae imaged from 2 to 10 hpi. Images were taken every 15 min, and the movie runs at 10 fps.

Supplementary Movie 4 *mpeg*^+^ phagocytes engulf *sox10*^+^ debris in lysolecithin-injected spinal cords. Lysolecithin was injected into 6 dpf *Tg(sox10:mrfp);Tg(mpeg1:egfp)* larvae imaged from 2 to 20 hpi. Images were taken every 10 min, and the movie runs at 10 fps.

## References

[B1] Acar G. Tanriover G. Demir N. Kayisli U. A. Sati G. L. Yaba A. (2004). Ultrastructural and immunohistochemical similarities of two distinct entities; multiple sclerosis and hereditary motor sensory neuropathy. *Acta Histochem.* 106 363–371. 10.1016/j.acthis.2004.08.004 15530551

[B2] Almeida R. G. Czopka T. Ffrench-Constant C. Lyons D. A. (2011). Individual axons regulate the myelinating potential of single oligodendrocytes in vivo. *Development* 138 4443–4450. 10.1242/dev.071001 21880787 PMC3177314

[B3] Arnett H. A. Mason J. Marino M. Suzuki K. Matsushima G. K. Ting J. P. (2001). TNF alpha promotes proliferation of oligodendrocyte progenitors and remyelination. *Nat. Neurosci.* 4 1116–1122. 10.1038/nn738 11600888

[B4] Azin M. Mirnajafi-Zadeh J. Javan M. (2015). Fibroblast growth factor-2 enhanced the recruitment of progenitor cells and myelin repair in experimental demyelination of rat hippocampal formations. *Cell J.* 17 540–456. 10.22074/cellj.2015.14 26464826 PMC4601875

[B5] Binari L. A. Lewis G. M. Kucenas S. (2013). Perineurial glia require Notch signaling during motor nerve development but not regeneration. *J. Neurosci.* 33 4241–4252. 10.1523/JNEUROSCI.4893-12.2013 23467342 PMC3668856

[B6] Bitsch A. Kuhlmann T. Da Costa C. Bunkowski S. Polak T. Brück W. (2000). Tumour necrosis factor alpha mRNA expression in early multiple sclerosis lesions: correlation with demyelinating activity and oligodendrocyte pathology. *Glia* 29 366–375. 10652446 10.1002/(sici)1098-1136(20000215)29:4<366::aid-glia7>3.0.co;2-y

[B7] Blakemore W. F. (1973). Demyelination of the superior cerebellar peduncle in the mouse induced by cuprizone. *J. Neurol. Sci.* 20 63–72. 10.1016/0022-510x(73)90118-4 4744511

[B8] Blakemore W. F. (1974). Remyelination of the superior cerebellar peduncle in old mice following demyelination induced by cuprizone. *J. Neurol. Sci.* 22 121–126. 10.1016/0022-510x(74)90059-8 4830551

[B9] Blakemore W. F. Eames R. A. Smith K. J. McDonald W. I. (1977). Remyelination in the spinal cord of the cat following intraspinal injections of lysolecithin. *J. Neurol. Sci.* 33 31–43. 10.1016/0022-510x(77)90179-4 903788

[B10] Blakemore W. F. Franklin R. J. (2008). Remyelination in experimental models of toxin-induced demyelination. *Curr. Top. Microbiol. Immunol.* 318 193–212. 10.1007/978-3-540-73677-6_8 18219819

[B11] Chiang C. S. Powell H. C. Gold L. H. Samimi A. Campbell I. L. (1996). Macrophage/microglial-mediated primary demyelination and motor disease induced by the central nervous system production of interleukin-3 in transgenic mice. *J. Clin. Invest.* 97 1512–1524. 10.1172/JCI118574 8617885 PMC507212

[B12] Chung A. Y. Kim P. S. Kim S. Kim E. Kim D. Jeong I. (2013). Generation of demyelination models by targeted ablation of oligodendrocytes in the zebrafish CNS. *Mol. Cells* 36 82–87. 10.1007/s10059-013-0087-9 23807048 PMC3887923

[B13] Copelman C. A. Diemel L. T. Gveric D. Gregson N. A. Cuzner M. L. (2001). Myelin phagocytosis and remyelination of macrophage-enriched central nervous system aggregate cultures. *J. Neurosci. Res.* 66 1173–1178. 10.1002/jnr.10026 11746450

[B14] Cunha M. I. Su M. Cantuti-Castelvetri L. Muller S. A. Schifferer M. Djannatian M. (2020). Pro-inflammatory activation following demyelination in required for myelin clearance and oligodendrogenesis. *J. Exp. Med.* 217:e20191390. 10.1084/jem.20191390 32078678 PMC7201919

[B15] Curado S. Stainier D. Y. Anderson R. M. (2008). Nitroreductase-mediated cell/tissue ablation in zebrafish: a spatially and temporally controlled ablation method with applications in developmental and regeneration studies. *Nat. Protoc.* 3 948–954. 10.1038/nprot.2008.58 18536643 PMC2705989

[B16] Czopka T. Ffrench-Constant C. Lyons D. A. (2013). Individual oligodendrocytes have only a few hours in which to generate new myelin sheaths in vivo. *Dev. Cell* 25 599–609. 10.1016/j.devcel.2013.05.013 23806617 PMC4013507

[B17] Czopka T. Lyons D. A. (2011). Dissecting mechanisms of myelinated axon formation using zebrafish. *Methods Cell Biol.* 105 25–62. 10.1016/b978-0-12-381320-6.00002-3 21951525

[B18] Davison J. M. Akitake C. M. Goll M. G. Rhee J. M. Gosse N. Baier H. (2007). Transactivation from Gal4-VP16 transgenic insertions for tissue-specific cell labeling and ablation in zebrafish. *Dev. Biol.* 304 811–824. 10.1016/j.ydbio.2007.01.033 17335798 PMC3470427

[B19] Domingues H. S. Portugal C. C. Socodato R. Relvas J. B. (2016). Oligodendrocyte, astrocyte, and microglia crosstalk in myelin development, damage, and repair. *Front. Cell Dev. Biol.* 4:71. 10.3389/fcell.2016.00071 27551677 PMC4923166

[B20] Doring A. Sloka S. Lau L. Mishra M. van Minnen J. Zhang X. (2015). Stimulation of monocytes, macrophages, and microglia by amphotericin B and macrophage colony-stimulating factor promotes remyelination. *J. Neurosci.* 35 1136–1148. 10.1523/JNEUROSCI.1797-14.2015 25609628 PMC6605538

[B21] Dousset V. Brochet B. Vital A. Gross C. Benazzouz A. Boullerne A. (1995). Lysolecithin-induced demyelination in primates: preliminary in vivo study with MR and magnetization transfer. *Am. J. Neuroradiol.* 16 225–231. 7726066 PMC8338338

[B22] Ellett F. Pase L. Hayman J. W. Andrianopoulos A. Lieschke G. J. (2011). mpeg1 promoter transgenes direct macrophage-lineage expression in zebrafish. *Blood* 117 e49–e56. 10.1182/blood-2010-10-314120 21084707 PMC3056479

[B23] Fang Y. Lei X. Li X. Chen Y. Xu F. Feng X. (2014). A novel model of demyelination and remyelination in a GFP-transgenic zebrafish. *Biol. Open* 4 62–68. 10.1242/bio.201410736 25527642 PMC4295166

[B24] Foote A. K. Blakemore W. F. (2005). Inflammation stimulates remyelination in areas of chronic demyelination. *Brain* 128 (Pt 3) 528–539. 10.1093/brain/awh417 15699059

[B25] Girard C. Bemelmans A. P. Dufour N. Mallet J. Bachelin C. Nait-Oumesmar B. (2005). Grafts of brain-derived neurotrophic factor and neurotrophin 3-transduced primate Schwann cells lead to functional recovery of the demyelinated mouse spinal cord. *J. Neurosci.* 25 7924–7933. 10.1523/JNEUROSCI.4890-04.2005 16135749 PMC6725455

[B26] Gregg C. Shikar V. Larsen P. Mak G. Chojnacki A. Yong V. W. (2007). White matter plasticity and enhanced remyelination in the maternal CNS. *J. Neurosci.* 27 1812–1823. 10.1523/JNEUROSCI.4441-06.2007 17314279 PMC6673564

[B27] Helker C. S. Schuermann A. Karpanen T. Zeuschner D. Belting H. G. Affolter M. (2013). The zebrafish common cardinal veins develop by a novel mechanism: lumen ensheathment. *Development* 140 2776–2786. 10.1242/dev.091876 23698350

[B28] Hill K. E. Zollinger L. V. Watt H. E. Carlson N. G. Rose J. W. (2004). Inducible nitric oxide synthase in chronic active multiple sclerosis plaques: distribution, cellular expression and association with myelin damage. *J. Neuroimmunol.* 151 171–179. 10.1016/j.jneuroim.2004.02.005 15145615

[B29] Hinks G. L. Franklin R. J. (2000). Delayed changes in growth factor gene expression during slow remyelination in the CNS of aged rats. *Mol. Cell Neurosci.* 16 542–556. 10.1006/mcne.2000.0897 11083917

[B30] Hirano A. Llena J. (2006). Fine structure of neuronal and glial processes in neuropathology. *Neuropathology* 26 1–7. 10.1111/j.1440-1789.2006.00647.x 16521474

[B31] Hirano A. Zimmermann H. M. Levine S. (1965). Fine structure of cerebral fluid accumulation: VI. Intracellular accumulation of fluid and Cryptococcal polysaccharide in oligodendroglia. *Arch. Neurol.* 12 189–196. 10.1001/archneur.1965.00460260079009 14237776

[B32] Huitinga I. van Rooijen N. de Groot C. J. Uitdehaag B. M. Dijkstra C. D. (1990). Suppression of experimental allergic encephalomyelitis in Lewis rats after elimination of macrophages. *J. Exp. Med.* 172 1025–1033. 10.1084/jem.172.4.1025 2145387 PMC2188611

[B33] Jeffery N. D. Blakemore W. F. (1995). Remyelination of mouse spinal cord axons demyelinated by local injection of lysolecithin. *J. Neurocytol.* 24 775–781. 10.1007/BF01191213 8586997

[B34] Jenkins S. J. Ruckerl D. Cook P. C. Jones L. H. Finkelman F. D. van Rooijen N. (2011). Local macrophage proliferation, rather than recruitment from the blood, is a signature of TH2 inflammation. *Science* 332 1284–1288. 10.1126/science.1204351 21566158 PMC3128495

[B35] Jung J. Cai W. Lee H. K. Pellegatta M. Shin Y. K. Jang S. Y. (2011). Actin polymerization is essential for myelin sheath fragmentation during wallerian degeneration. *J. Neurosci.* 31 2009–2015. 10.1523/jneurosci.4537-10.2011 21307239 PMC3071261

[B36] Karttunen M. J. Czopka T. Goedhart M. Early J. J. Lyons D. A. (2017). Regeneration of myelin sheaths of normal length and thickness in the zebrafish CNS correlates with growth of axons in caliber. *PLoS One* 12:e0178058. 10.1371/journal.pone.0178058 28542521 PMC5444792

[B37] Karttunen M. J. Lyons D. A. (2019). A drug-inducible transgenic zebrafish model for myelinating glial cell ablation. *Methods Mol. Biol.* 1936 227–238. 10.1007/978-1-4939-9072-6_13 30820902

[B38] Kaufmann W. Bolon B. Bradley A. Butt M. Czasch S. Garman R. H. (2012). Proliferative and nonproliferative lesions of the rat and mouse central and peripheral nervous systems. *Toxicol. Pathol.* 40 (Suppl. 4) 87S–157S. 10.1177/0192623312439125 22637737

[B39] Kearns C. A. Ravanelli A. M. Cooper K. Appel B. (2015). Fbxw7 limits myelination by inhibiting mTOR signaling. *J. Neurosci.* 35 14861–14871. 10.1523/jneurosci.4968-14.2015 26538655 PMC4635133

[B40] Keough M. B. Jensen S. K. Yong V. W. (2015). Experimental demyelination and remyelination of murine spinal cord by focal injection of lysolecithin. *J. Vis. Exp.* 97:e52679. 10.3791/52679 25867716 PMC4401378

[B41] Kimmel C. B. Ballard W. W. Kimmel S. R. Ullmann B. Schilling T. F. (1995). Stages of embryonic development of the zebrafish. *Dev. Dyn.* 203 253–310. 10.1002/aja.1002030302 8589427

[B42] Kotter M. R. Setzu A. Sim F. J. Van Rooijen N. Franklin R. J. M. (2001). Macrophage depletion impairs oligodendrocyte remyelination following lysolecithin-induced demyelination. *Glia* 35 204–212. 10.1002/glia.1085 11494411

[B43] Kotter M. R. Zhao C. van Rooijen N. Franklin R. J. M. (2005). Macrophage-depletion induced impairment of experimental CNS remyelination is associated with a reduced oligodendrocyte progenitor cell response and altered growth factor expression. *Neurobiol. Dis.* 18 166–175. 15649707 10.1016/j.nbd.2004.09.019

[B44] Kucenas S. Takada N. Park H.-C. C. Woodruff E. Broadie K. Appel B. (2008). CNS-derived glia ensheath peripheral nerves and mediate motor root development. *Nat. Neurosci.* 11 143–151. 18176560 10.1038/nn2025 PMC2657597

[B45] Kucharova K. Chang Y. Boor A. Yong V. W. Stallcup W. B. (2011). Reduced inflammation accompanies diminished myelin damage and repair in the NG2 null mouse spinal cord. *J. Neuroinflamm.* 8:158. 10.1186/1742-2094-8-158 22078261 PMC3229456

[B46] Lampron A. Larochelle A. Laflamme N. Préfontaine P. Plante M. M. Sánchez M. G. (2015). Inefficient clearance of myelin debris by microglia impairs remyelinating processes. *J. Exp. Med.* 212 481–495. 10.1084/jem.20141656 25779633 PMC4387282

[B47] Lassmann H. Brück W. Lucchinetti C. (2001). Heterogeneity of multiple sclerosis pathogenesis: implications for diagnosis and therapy. *Trends Mol. Med.* 7 115–121. 10.1016/s1471-4914(00)01909-2 11286782

[B48] Lau L. W. Keough M. B. Haylock-Jacobs S. Cua R. Doring A. Sloka S. (2012). Chondroitin sulfate proteoglycans in demyelinated lesions impair remyelination. *Ann. Neurol.* 72 419–432. 10.1002/ana.23599 23034914

[B49] Locatelli G. Baggiolini A. Schreiner B. Palle P. Waisman A. Becher B. (2015). Mature oligodendrocytes actively increase in vivo cytoskeletal plasticity following CNS damage. *J. Neuroinflamm.* 12:62. 10.1186/s12974-015-0271-2 25889302 PMC4404661

[B50] Mason J. L. Suzuki K. Chaplin D. D. Matsushima G. K. (2001). Interleukin-1beta promotes repair of the CNS. *J. Neurosci.* 21 7046–7052. 10.1523/jneurosci.21-18-07046.2001 11549714 PMC6762979

[B51] Matsumoto Y. Ohmori K. Fujiwara M. (1992). Microglial and astroglial reactions to inflammatory lesions of experimental autoimmune encephalomyelitis in the rat central nervous system. *J. Neuroimmunol.* 37 23–33. 10.1016/0165-5728(92)90152-b 1372328

[B52] McGraw H. F. Snelson C. D. Prendergast A. Suli A. Raible D. W. (2012). Postembryonic neuronal addition in zebrafish dorsal root ganglia is regulated by Notch signaling. *Neural Dev.* 7:23. 10.1186/1749-8104-7-23 22738203 PMC3438120

[B53] Mensch S. Baraban M. Almeida R. Czopka T. Ausborn J. El Manira A. (2015). Synaptic vesicle release regulates myelin sheath number of individual oligodendrocytes in vivo. *Nat. Neurosci.* 18 628–630. 10.1038/nn.3991 25849985 PMC4427868

[B54] Miller R. H. Fyffe-Maricich S. L. (2010). Restoring the balance between disease and repair in multiple sclerosis: insights from mouse models. *Dis. Model Mech.* 3 535–539. 10.1242/dmm.001958 20647413 PMC2931531

[B55] Münzel E. J. Becker C. G. Becker T. Williams A. (2014). Zebrafish regenerate full thickness optic nerve myelin after demyelination, but this fails with increasing age. *Acta Neuropathol. Commun.* 2:77. 10.1186/s40478-014-0077-y 25022486 PMC4164766

[B56] Nait-Oumesmar B. Decker L. Lachapelle F. Avellana-Adalid V. Bachelin C. Van Evercooren A. B. (1999). Progenitor cells of the adult mouse subventricular zone proliferate, migrate and differentiate into oligodendrocytes after demyelination. *Eur. J. Neurosci.* 11 4357–4366. 10.1046/j.1460-9568.1999.00873.x 10594662

[B57] Nawaz S. Sánchez P. Schmitt S. Snaidero N. Mitkovski M. Velte C. (2015). Actin filament turnover drives leading edge growth during myelin sheath formation in the central nervous system. *Dev. Cell* 34 139–151. 26166299 10.1016/j.devcel.2015.05.013 PMC4736019

[B58] Ousman S. S. David S. (2000). Lysophosphatidylcholine induces rapid recruitment and activation of macrophages in the adult mouse spinal cord. *Glia* 30 92–104. 10696148

[B59] Palha N. Guivel-Benhassine F. Briolat V. Lutfalla G. Sourisseau M. Ellett F. (2013). Real-time whole-body visualization of Chikungunya Virus infection and host interferon response in zebrafish. *PLoS Pathog.* 9:e1003619. 10.1371/journal.ppat.1003619 24039582 PMC3764224

[B60] Pisharath H. Parsons M. J. (2009). “Nitroreductase-mediated cell ablation in transgenic Zebrafish embryos,” in *Zebrafish: Methods and Protocols*, eds Lieschke G. J. Oates A. C. Kawakami K. (Totowa, NJ: Humana Press), 133–143. 10.1007/978-1-60327-977-2_9 19378102

[B61] Pisharath H. Rhee J. M. Swanson M. A. Leach S. D. Parsons M. J. (2007). Targeted ablation of beta cells in the embryonic zebrafish pancreas using *E. coli* nitroreductase. *Mech. Dev.* 124 218–229. 10.1016/j.mod.2006.11.005 17223324 PMC2583263

[B62] Prendergast A. Linbo T. H. Swarts T. Ungos J. M. McGraw H. F. Krispin S. (2012). The metalloproteinase inhibitor Reck is essential for zebrafish DRG development. *Development* 139 1141–1152. 10.1242/dev.072439 22296847 PMC3283124

[B63] Quinn M. T. Parthasarathy S. Steinberg D. (1988). Lysophosphatidylcholine: a chemotactic factor for human monocytes and its potential role in atherogenesis. *Proc. Natl. Acad. Sci. U.S.A.* 85 2805–2809. 10.1073/pnas.85.8.2805 3357891 PMC280088

[B64] Raivich G. Banati R. (2004). Brain microglia and blood-derived macrophages: molecular profiles and functional roles in multiple sclerosis and animal models of autoimmune demyelinating disease. *Brain Res. Rev.* 46 261–281. 10.1016/j.brainresrev.2004.06.006 15571769

[B65] Ransohoff R. M. (2012). Animal models of multiple sclerosis: the good, the bad and the bottom line. *Nat. Neurosci.* 15 1074–1077. 10.1038/nn.3168 22837037 PMC7097342

[B66] Rawji K. S. Yong V. W. (2013). The benefits and detriments of macrophages/microglia in models of multiple sclerosis. *Clin. Dev. Immunol.* 2013:948976. 10.1155/2013/948976 23840244 PMC3694375

[B67] Remington L. T. Babcock A. A. Zehntner S. P. Owens T. (2007). Microglial recruitment, activation, and proliferation in response to primary demyelination. *Am. J. Pathol.* 170 1713–1724. 10.2353/ajpath.2007.060783 17456776 PMC1854965

[B68] Rinner W. A. Bauer J. Schmidts M. Lassmann H. Hickey W. F. (1995). Resident microglia and hematogenous macrophages as phagocytes in adoptively transferred experimental autoimmune encephalomyelitis: an investigation using rat radiation bone marrow chimeras. *Glia* 14 257–266. 10.1002/glia.440140403 8530183

[B69] Rose J. W. Hill K. E. Watt H. E. Carlson N. G. (2004). Inflammatory cell expression of cyclooxygenase-2 in the multiple sclerosis lesion. *J. Neuroimmunol.* 149 40–49. 10.1016/j.jneuroim.2003.12.021 15020063

[B70] Sahel A. Ortiz F. C. Kerninon C. Maldonado P. P. Angulo M. C. Nait-Oumesmar B. (2015). Alteration of synaptic connectivity of oligodendrocyte precursor cells following demyelination. *Front. Cell Neurosci.* 9:77. 10.3389/fncel.2015.00077 25852473 PMC4362325

[B71] Samanta J. Salzer J. L. (2015). Myelination: actin disassembly leads the way. *Dev. Cell* 34 129–130. 10.1016/j.devcel.2015.07.006 26218317 PMC4970517

[B72] Schönrock L. M. Kuhlmann T. Adler S. Bitsch A. Brück W. (1998). Identification of glial cell proliferation in early multiple sclerosis lesions. *Neuropathol. Appl. Neurobiol.* 24 320–330. 10.1046/j.1365-2990.1998.00131.x 9775398

[B73] Stys P. K. Zamponi G. W. van Minnen J. Geurts J. J. (2012). Will the real multiple sclerosis please stand up? *Nat. Rev. Neurosci.* 13 507–514. 10.1038/nrn3275 22714021

[B74] Travnickova J. Tran Chau V. Julien E. Mateos-Langerak J. Gonzalez C. Lelièvre E. (2015). Primitive macrophages control HSPC mobilization and definitive haematopoiesis. *Nat. Commun.* 6:6227. 10.1038/ncomms7227 25686881

[B75] Triarhou L. C. Herndon R. M. (1985). Effect of macrophage inactivation on the neuropathology of lysolecithin-induced demyelination. *Br. J. Exp. Pathol.* 66 293–301. 4005147 PMC2041059

[B76] Waller A. (1851). Experiments on the section of the Glosso-pharyngeal and hypoglossal nerves of the frog, and observations of the alterations produced thereby in the structure of their primitive fibres. *Edinb. Med. Surg. J.* 76 369–376. PMC5929074 30332247

[B77] Wiedenmann J. Ivanchenko S. Oswald F. Schmitt F. Röcker C. Salih A. (2004). EosFP, a fluorescent marker protein with UV-inducible green-to-red fluorescence conversion. *Proc. Natl. Acad. Sci. U.S.A.* 101 15905–15910. 10.1073/pnas.0403668101 15505211 PMC528746

[B78] Zuchero J. B. Fu M. M. Sloan S. A. Ibrahim A. Olson A. Zaremba A. (2015). CNS myelin wrapping is driven by actin disassembly. *Dev. Cell* 34 152–167. 10.1016/j.devcel.2015.06.011 26166300 PMC4519368

